# Poly(Oxyethylene)-Amidoamine Based Gemini Cationic Surfactants for Oilfield Applications: Effect of Hydrophilicity of Spacer Group

**DOI:** 10.3390/ma13051046

**Published:** 2020-02-26

**Authors:** S. M. Shakil Hussain, Ahmad Mahboob, Muhammad Shahzad Kamal

**Affiliations:** Center for integrative Petroleum Research, King Fahd University of Petroleum & Minerals, Dhahran 31261, Saudi Arabia; smshakil@kfupm.edu.sa (S.M.S.H.); ahmad.mahboob@kfupm.edu.sa (A.M.)

**Keywords:** poly(oxyethylene), synthesis, thermal, surface, oilfield

## Abstract

Thermal stability, salt tolerance, and solubility in normal and high salinity brine are the major requirements for any surfactant designed for oilfield applications because the surfactant stays in a non-ambient environment inside the reservoir for a long period of time. Herein, a series of new gemini cationic surfactants (GSs) with varying spacer hydrophilicity were synthesized and elucidated using MALDI-ToF-MS, NMR (1H, 13C), as well as FTIR spectroscopy. GSs found to be soluble in normal as well as high salinity brine and aqueous stability tests revealed that GSs possess the ability to retain their structural integrity at high salinity and high temperature conditions because no suspension formation or precipitation was detected in the oven aged sample of GSs at 90 °C for 30 days. Thermal gravimetric analysis displayed a higher decomposition temperature than the real reservoir temperature and the GS with a secondary amine spacer exhibited high heat stability. The significant reduction in surface tension and critical micelle concentration was observed using 1 M NaCl solution in place of deionized water. The difference in surface tension and critical micelle concentration was insignificant when the 1 M NaCl solution was replaced with seawater. The synthesized surfactants can be utilized for oilfield applications in a challenging high temperature high salinity environment.

## 1. Introduction

Surfactants are extensively applied in the petroleum industry such as enhanced oil recovery (EOR) [1,2,3,4,5], fracking fluid [6], swelling prevention [7], reservoir stimulation [8], corrosion inhibitor [9], drag reduction [10], and drilling mud [11,12]. Surfactants minimize the interfacial tension (IFT) of crude oil and aqueous phase as well as alter the wettability of oilfield rocks [13,14,15]. There are different classes of surfactants such as cationic, anionic, and zwitterionic as well as nonionic, and the choice of an appropriate class exclusively relies on the type of oilfield. As an example, negatively charged surfactants are avoided in carbonate reservoirs and positively charged surfactants are avoided in sandstone reservoirs because of their high adsorption.

Gemini surfactants (GSs) belong to a novel class that contains more than one conventional surfactant joined by the spacer group. GSs display unique chemical, physical, and aggregation morphologies compared to those of single head single tail surfactants. The important features of GSs are smaller critical micelle concentration (CMC), good solubilizing capability, antibacterial activity, required low quantity, and unique aggregation behavior [16]. Due to these distinctive properties, GSs have found application in the oilfield industry.

The nature of the spacer group is perhaps the most indispensable factor that controls the chemical and physical properties of the GSs because the spacer can handle hydrophobic interaction and constrain the repulsion of the hydrophilic headgroup. The spacer group can be short [17,18,19], long [20], hydrophilic [21], hydrophobic [22], rigid [23], flexible [24], aromatic [25], etc. Zhou et al. studied the swelling inhibition properties of GSs with different lengths of the lipophilic tail and hydrophilic spacer group and observed that the spacer could increase the interaction with montmorillonite clay by hydrogen bonding, but an overlong spacer could lead to poor inhibition performance [26]. Mao et al. reported the synthesis of a gemini cationic surfactant with a methylamine and epoxy chloropropane spacer for thickening material and applied in fracturing fluid applications [27]. They claimed the synthesized material exhibited good temperature stability and in the following report, they developed an efficient method for recycling the same surfactant gel from the flowback fluid [28]. Pal and co-workers investigated the influence of the length of the spacer in a gemini cationic surfactant for EOR. They observed an increase in oil recovery efficiency by increasing the length of the spacer in the order of 29.83%, 31.73%, 33.83%, and 34.55% by using 3, 4, 5, and 6 carbons in the spacer group, respectively [19]. Migahed et al. reported the synthesis of two gemini cationic surfactants containing different spacer lengths as anti-corrosion for X-65 steel dissolution in oilfields produced water under sweet conditions [29]. They observed that the surfactant with a shorter spacer length exhibited better inhibitive properties than the surfactant with a comparatively larger spacer length. Similarly, Cao et al. observed lower CMC values of the synthesized gemini surfactant containing four ethoxy units in the spacer group compared with the corresponding gemini surfactant with eight ethoxy units in the spacer group [30].

Aqueous stability or solubility of the surfactant is an essential parameter for a required formulation, which expressively affects the propagation of the surfactant inside the reservoir. Commercial surfactants suffer due to low aqueous stability or solubility in high salinity reservoir brine (≈22,000 ppm). For example, alkylbenzene sulfonates show cloudiness when they encounter alkali and avoid the requirement of the transparent solution in the surfactant formulation stage [31]. Surfactants used in oilfield applications must be chemically stable in high temperatures and high salinity environments for several days to months because these harsh conditions significantly affect the chemical stability of the surfactant inside the reservoir.

The objectives of this work include (1) synthesis of three new gemini cationic surfactants (GSs) containing a diethyl ether spacer (**g1**), secondary amine spacer (**g2**), isopropyl alcohol spacer (**g3**), and the spectral characterization with the aid of MALDI-ToF-MS, FTIR, 1H, and 13C NMR; (2) aqueous stability tests of the GSs for 30 days at 90 °C in seawater (SW), formation water (FW), and distilled water (DW); (3) the heat resistance experiment of GSs using thermal gravimetric analysis (TGA); and (4) the influence of different hydrophilic spacer groups onto the aggregation behavior. The chemical structures were sensibly planned in order to acquire certain properties. For example, the GSs with a hydrophilic spacer showed a lower CMC in comparison with the corresponding GSs containing a hydrophobic spacer group [32]. The solubility of the GSs in normal and high saline water was achieved by adding a sufficient number of ethoxy units [33].

## 2. Materials and Synthesis

### 2.1. Materials

Bis(2-chloroethyl)amine hydrochloride (98%), 3-(dimethylamino)-1-propylamine (99%), bis(2-bromoethyl) ether (95%), glycolic acid ethoxylate lauryl ether (average M_n_ ~690), NaF (≥99%), 1,3-dichloro-2-propanol (98%), and Al_2_O_3_ (99.99%) were received from Aldrich. The SW and FW were formed through the addition of a specific amount of MgCl_2_, CaCl_2_, NaHCO_3_, Na_2_SO_4_, and NaCl (Table 5), and the material was received from Panreac.

### 2.2. Structure Determination

The NMR study was done using the Bruker machine (500 MHz, Jeol, Tokyo, Japan) by using tetramethylsilane (TMS) as an internal standard. The NMR (proton and carbon-13) was conducted in the deuterated solvent and the graph was plotted in ppm. The FTIR study was performed on the 16F model of the Perkin-Elmer instrument (Waltham, MA, USA) and the FTIR graph was plotted in cm^−1^. The MALDI-ToF-MS analysis was performed using a Bruker SolariX XR instrument (Bruker, Billerica, MA, USA).

### 2.3. Solubility Tests

Five wt % of **g1**, **g2**, and **g3** was solubilized in SW, FW, and DW, followed by oven aging for 30 days at 90 °C and the solubility was visually detected. Stability was confirmed by NMR.

### 2.4. Thermal Gravimetric Analysis (TGA)

A SDT Q600 machine (TA instrument, New Castle, DE, USA) was employed for TGA measurements. Tests were done in a continuous heating rate (20 °C /min) and a stable flow of nitrogen (100 mL/min) with a 30 °C to 500 °C temperature interval.

### 2.5. Surface Tension Experiments

Surface tension experiments of **g1**, **g2**, and **g3** were done using a force tensiometer (Sigma 702, Biolin Scientific, Gothenburg, Sweden) using the Wilhelmy plate method. Measurements were performed at 25 ± 0.1 °C with proper cleaning of the plate. The surface tension value of DW was used as a standard. The surface tension at each concentration was repeated five times. The surface tension standard deviation ranged from ±0.0390 mN/m to ±0.0999 for different readings.

### 2.6. Synthesis

Synthesis of **g1**

**Scheme 1:**
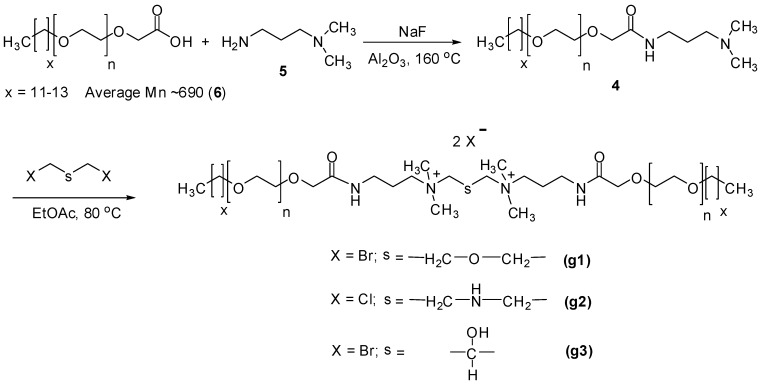
Synthesis of gemini surfactants having various hydrophilic spacer group.

**g1** was synthesized by the amidation of glycolic acid ethoxylate lauryl ether (**6**) (100 g, 144.93 mmol) with 3-(dimethylamino)-1-propylamine **5** (29.62 g, 289.85 mmol) using NaF (0.61 g, 14.49 mmol) in a 500 mL flask up to 8 h at 160 °C in an argon atmosphere (Scheme 1). The resulting water of the reaction was absorbed using Al_2_O_3_. After 8 h, additional compound **5** (22.21 g, 217.39 mmol) was injected with continued stirring for an extra 6 h. After that, the leftover compound **5** was vaporized through reduced pressure and the solid NaF was filtered. The resulting product was dried to obtain compound **4** [34]. In the following step, compound **4** (25.0 g, 32.30 mmol) was treated with bis(2-bromoethyl) ether (3.26 g, 14.04 mmol) using dried EtOH (5 mL) for 48 h at 80 °C. Eventually, the solvent was extracted and the column chromatography was conducted by an ethanol-based mobile phase to acquire **g1** in a gel-like material [35].

The **g2** and **g3** were also synthesized using the same procedure respectively.

## 3. Results and Discussion

Scheme 1 shows the synthesis of gemini surfactants (**g1**, **g2**, and **g3**). 3-(dimethylamino)-1-propylamine (**5**) was stirred with glycolic acid ethoxylate lauryl ether (**6**) using NaF at 160 °C. Resulting compound **4** was separately reacted with bis(2-bromoethyl) ether, bis(2-chloroethyl)amine hydrochloride, and 1,3-dibromo-2-propanol to achieve the related **g1**, **g2**, and **g3**, respectively. Monomer formation is the expected side product of the reaction.

### 3.1. Interpretation of the GS Structure

**Figure 1:**
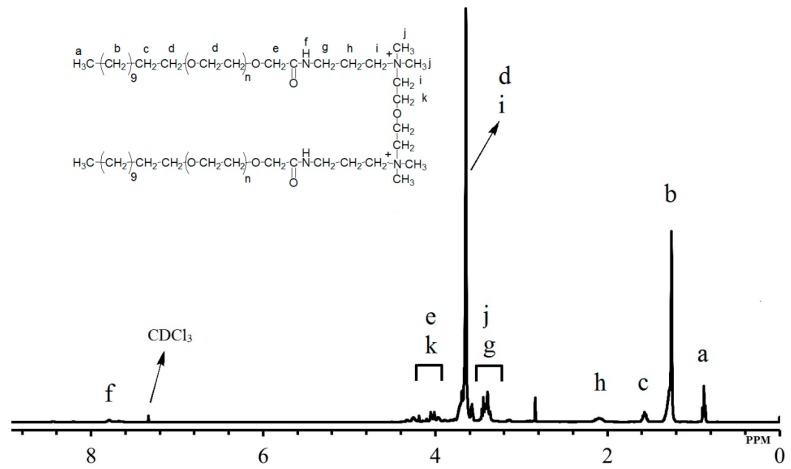
1H NMR spectra and peak assignment of **g1**.

The **g1**, **g2**, and **g3** structures were identified using MALDI-ToF-MS, NMR, and FTIR. We emphasized the structure identification of **g1** here as an example. Rendering the 1H NMR data of **g1** (Table 1, Figure 1), the peaks at δ 0.88 ppm and δ 1.26 ppm could be referred to the CH_3_ and CH_2_ groups in the lipophilic tail, respectively. The signal at δ 3.39 ppm could be assigned to two methyl groups of the positively charged ammonium head [N^+^(CH_3_)_2_]. Multiple signals of ethoxy were detected as an overlapped peak at δ 3.61–3.70 ppm. The broad peak at δ 7.79 ppm could be allocated to an amide group [NH(CO)].

**Figure 2:**
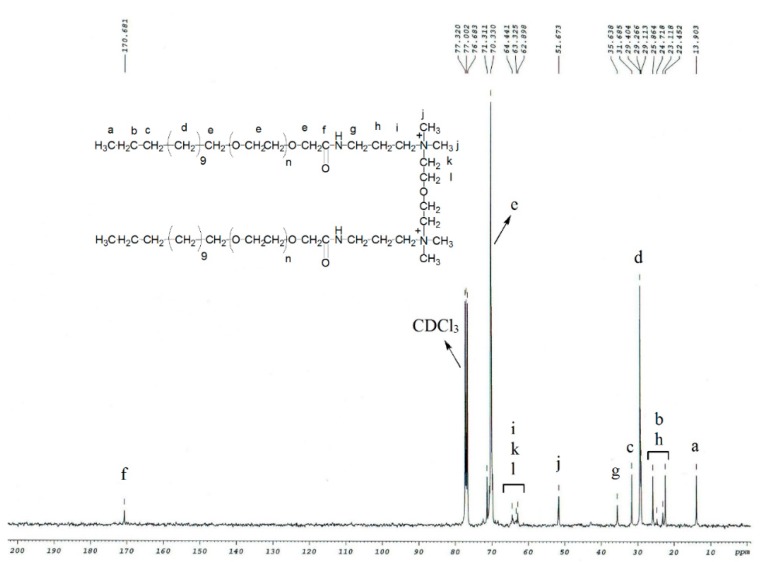
13C NMR spectra and peak assignment of **g1**.

Rendering the 13C NMR graph of **g1** (Table 2, Figure 2), the signals at δ 13.9, 25.9, 31.7, and 29.1–29.4 ppm could be assigned to the hydrocarbon of the lipophilic tail. The peak at δ 51.7 ppm could be coupled with the two CH_3_ groups of the positively charged ammonium head [–N^+^(CH_3_)_2_]. The signals detected at δ 62.9, 63.3, and 64.4 ppm could be related to two methylene units of the spacer and one methylene unit next to ammonium head [–O–CH_2_–CH_2_–N^+^-CH_2_–(CH_3_)_2_], respectively. The multiple peaks of methylene units [–O–CH_2_–CH_2_–] were observed at δ 69.0–71.5 ppm. The signal detected at δ 170.7 ppm could be linked with the carbonyl carbon of amide [CH_2_CONH].

**Figure 3:**
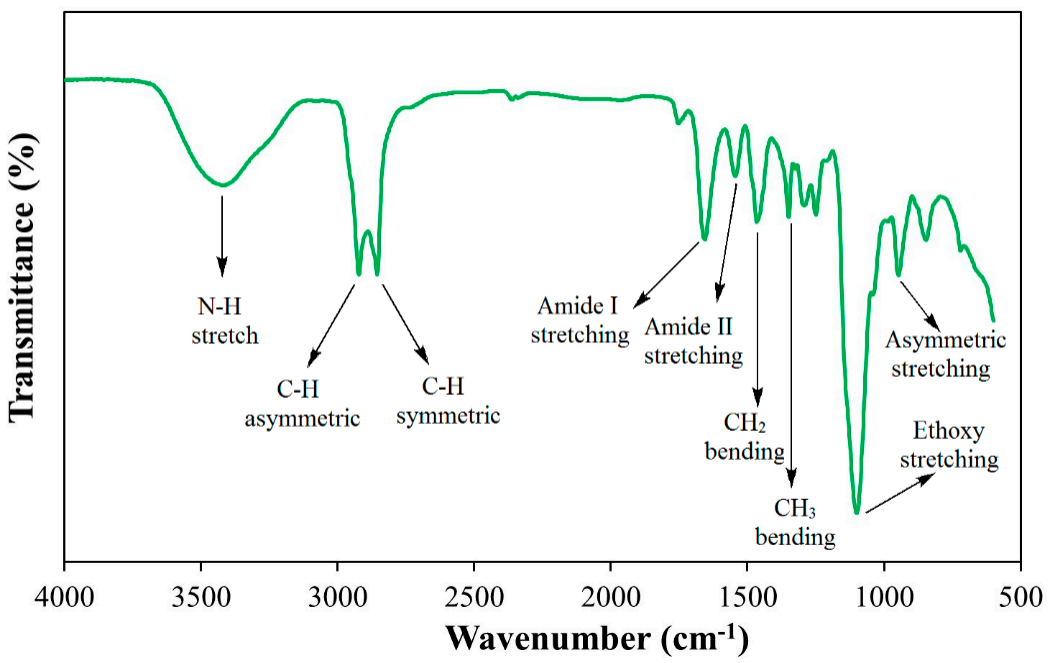
Infrared spectra and peak assignment of **g1.**

Rendering the FTIR spectra of **g1** (Table 3, Figure 3), a broad adsorption peak at 3424/cm corresponded to the NH stretch. Two adsorption bands at 2922/cm and 2855/cm could be assigned to the CH_2_ asymmetric and symmetric motion, respectively. Two adsorption bands at 1656/cm, and 1544/cm could be linked to the amide (I) and amide (II) stretching, respectively [38]. The ethoxy units were identified by the adsorption signals at 1100 cm^−1^.

**Figure 4:**
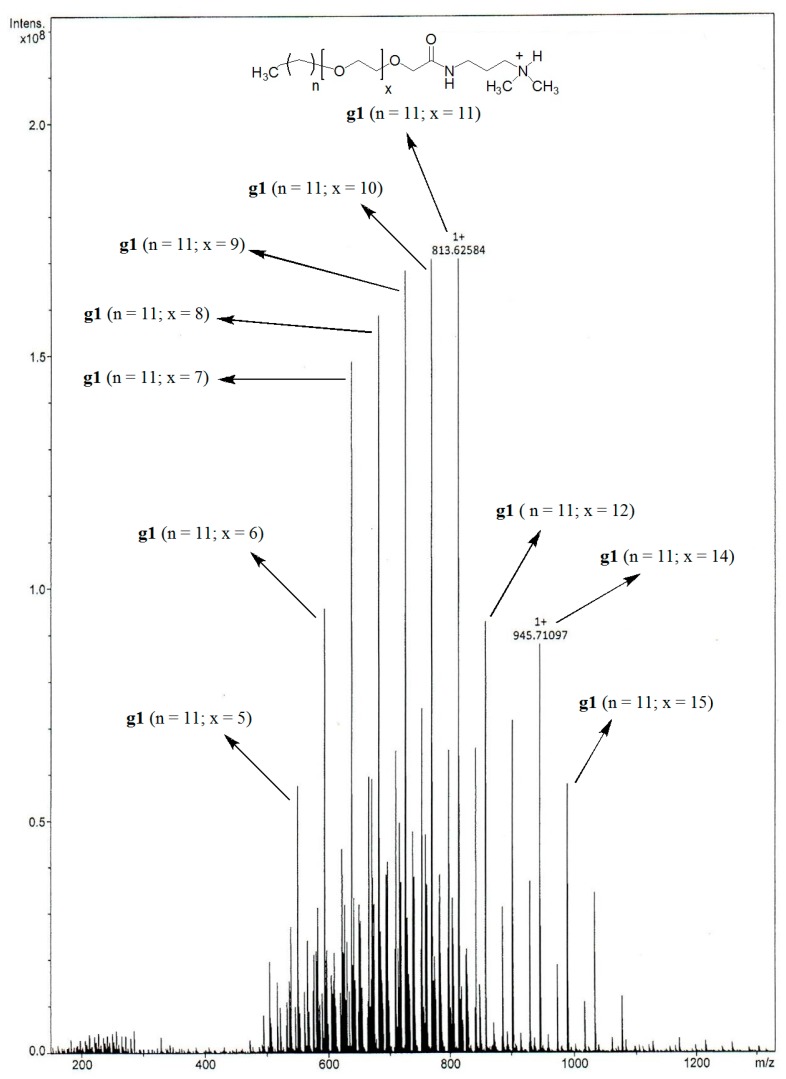
MALDI-ToF-MS spectra and peak assignment of **g1.**

Usually, FTIR and NMR data are adequate to determine the chemical structure; however, for surfactants such as **g1**–**g3**, which contain high molecular weight along with a distribution of ethoxy groups, an additional technique such as MALDI-ToF-MS is important to acquire insights into the chemical structure of a gemini surfactant. Rendering the MALDI-ToF-MS of **g1** (Table 4, Figure 4), the base peak was detected at *m*/*z* 813.63. This may be attributed to the heterolytic bond breaking between the terminal carbon of the spacer and the quaternary-N promoted by the ether oxygen of the spacer group, which leads to the conclusion that **g1** with x = 11 and *n* = 11 is the chief component (Figure 4). The following peaks after and before the base peak in the MALDI-ToF-MS spectra indicate the distribution of ethoxy groups because the mass difference of these peaks was *m*/*z* 44, which is a mass of one ethoxy group. For example, the peak of *m*/*z* 769.60 was assigned to a molecular mass of **g1** containing 10 ethoxy units (x = 10, *n* = 11) and the peak of *m*/*z* 725.57 referred to a molecular mass of **g1** having nine ethoxy units (x = 9, *n* = 11). Similarly, the signals of *m*/*z* 681.54 were assigned to a molecular mass of **g1** containing eight ethoxy units (x = 8, *n* = 11) and the peak of *m*/*z* 637.51 referred to a molecular mass of **g1** with seven ethoxy units (x = 7, *n* = 11). On the other hand, the peak of *m*/*z* 857.65 was assigned to a molecular mass of **g1** containing 12 ethoxy units (x = 12, *n* = 11) and the peak of *m*/*z* 901.68 referred to a molecular mass of **g1** with 13 ethoxy units (x = 13, *n* = 11). Likewise, the signals of *m*/*z* 945.71 was assigned to a molecular mass of **g1** containing 14 ethoxy units (x = 14, *n* = 11) and the peak of *m*/*z* 989.74 referred to a molecular mass of **g1** with 15 ethoxy units (x = 15, *n* = 11).

In general, the MALDI-ToF-MS, FTIR, and NMR results are in agreement with the chemical structure of **g1**.

### 3.2. Aqueous Stability and Solubility

**Figure 5:**
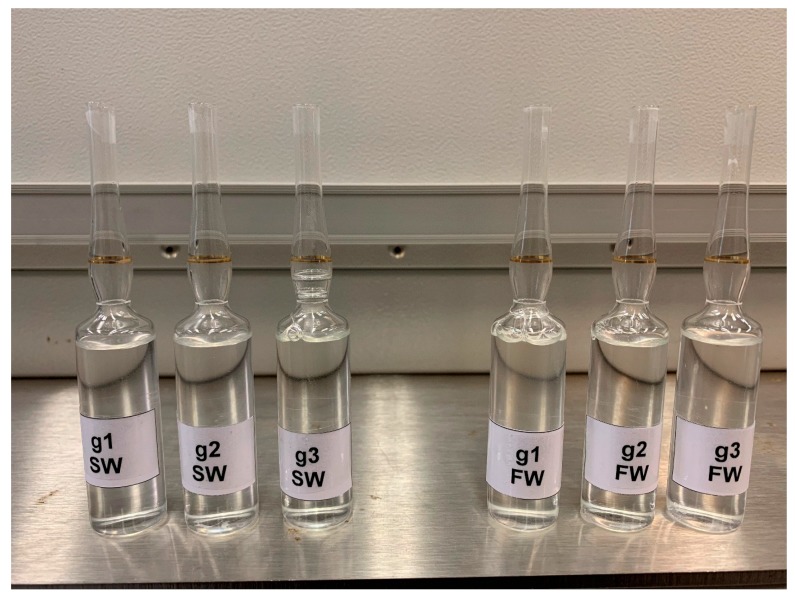
Snapshot of **g1**, **g2**, and **g3** solutions in SW and FW.

Surfactant tolerance in the presence of formation ions as well as solubility at high saline condition are the basic requirements for any new surfactant designed for oilfield applications. The synthesized **g1**, **g2**, and **g3** displayed excellent solubility in deionized and saltwater because of the polyethoxylates present in the chemical structure (Figure 5) [33]. The aqueous stability and heat resistance experiments were done by solubilizing **g1, g2**, and **g3** in SW, FW, and DW, followed by oven aging for 30 days at 90 °C. The salt concentration in the laboratory prepared FW and SW is shown in Table 5. Usually, solubility increases with an increase in temperature; however, temperature showed a negative impact on aqueous stability. The synthesized gemini surfactants (**g1**, **g2**, and **g3**) exhibited excellent solubility in normal as well as high saltwater and the transparent solution of the oven aged samples at 90 °C for 30 days revealed that the **g1**, **g2**, and **g3** are stable in normal and high saltwater because less stable and less soluble gemini surfactants tend to show precipitation or suspension formation under these harsh conditions. We further investigated the effect of heat on the chemical structure of the surfactants by doing 1H NMR analysis of the oven aged samples. As depicted in Figure S1 (Supplementary Materials), the 1H NMR spectra of the before aging and after aging samples of **g1** matched, which further confirmed the stability of the surfactant under harsh conditions.

### 3.3. Thermal Gravimetric Analysis (TGA)

**Figure 6:**
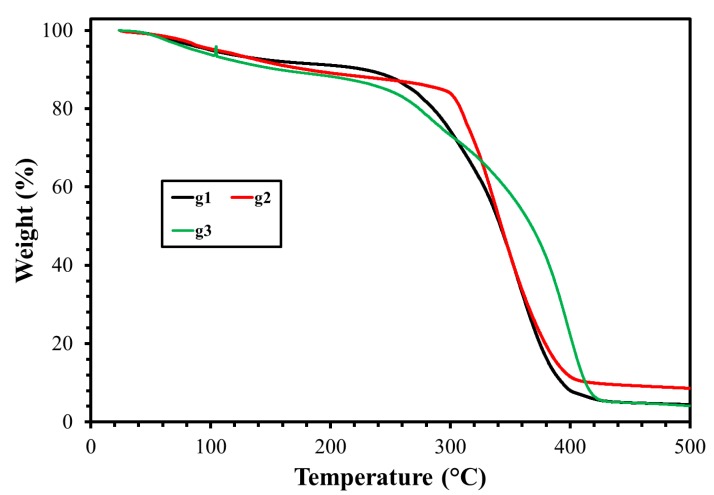
Thermal gravimetric analysis of **g1**, **g2**, and **g3** with different hydrophilicity of the spacer.

Surfactant heat stability is another important factor for any new surfactant designed for oilfield applications because the surfactant stays in a non-ambient environment inside the reservoir for a long period of time. Therefore, thermal decomposition studies for **g1**, **g2**, and **g3** were conducted using TGA. TGA analysis is only used to assess short-term thermal stability. However, for long-term thermal analysis, surfactants were aged at reservoir conditions for a specific time. After the aging period, thermal decomposition was assessed using visual observations, FTIR, and NMR. The TGA plot of weight % of **g1**, **g2**, and **g3** versus temperature is depicted in Figure 6. The first 10–15% weight loss of **g1**, **g2**, and **g3** was mainly due to the loss of moisture and impurities. The second step showed a big slope at 268 °C (**g1**), 302 °C (**g2**), and 260 °C (**g3**), exhibiting the influence of heat resulting in the structure degradation of **g1**, **g2**, and g3, respectively. All three surfactants showed almost identical degradation patterns; however, the GS with a secondary amine spacer (**g2**) exhibited comparatively higher heat stability. Overall, it was established that the synthesized gemini surfactants displayed a higher heat stability than the actual reservoir temperature (≥90 °C) and the hydrophilic nature of the spacer group showed a significant effect toward the thermal stabilities of the GSs.

### 3.4. Surface Tension Measurements

The CMC was measured with the aid of log surfactant molar concentration (C) vs. surface tension (γ) at 25 °C. The occupied minimum area of surfactant (A_min_) and the maximum surface access (г_max_) was investigated by the Gibbs equation [39].

(1) $${г}_{max}=-\frac{1}{nRT}{\left(\frac{d\gamma }{dlnC}\right)}_{T}$$

(2) $$A_{min}=10^{18}/N_{A}{г}_{max}$$

where dγ/dlnC is the gradient; R is the gas constant; T is the temperature; and N_A_ is Avogadro’s number [40].

**Figure 7:**
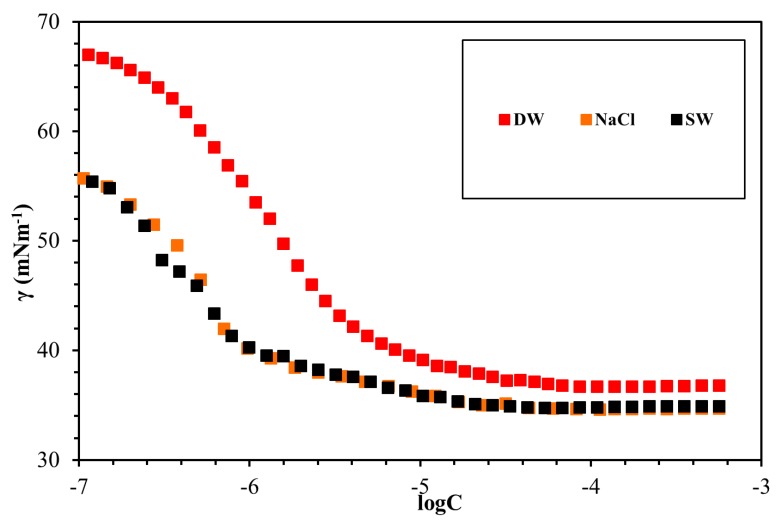
The surface tension of **g1** at different salinities.

**Figure 8:**
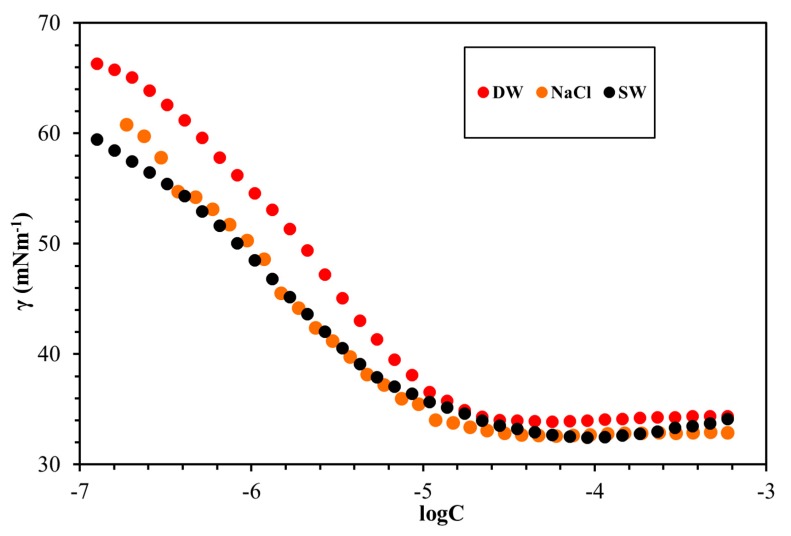
The surface tension of **g2** at different salinities.

**Figure 9:**
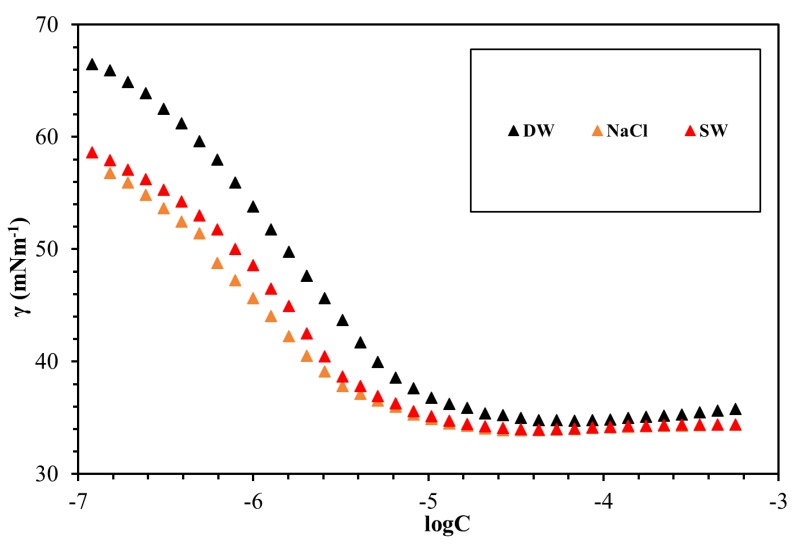
The surface tension of **g3** at different salinities.

Figure 7, Figure 8 and Figure 9 display the surface tension values of **g1**, **g2**, and **g3**, respectively. The surface tension of GSs was reduced by adding surfactant until it reached the CMC. Figure 7 highlights the surface tension of **g1** in DW, NaCl, and SW, and it was observed that the CMC decreased with the increasing salinity of the surfactant solution (DW ˃ NaCl) [41]. Likewise, surface tension at CMC (γ_CMC_) also decreased with increasing salinity of the surfactant solution (DW ˃ NaCl). However, no significant difference in the CMC and γ_CMC_ was noticed between NaCl and the seawater solution (NaCl ≈ SW), which may be due to no significant salinity difference between NaCl and seawater (Table 6). This behavior was consistent for all three types of GSs (**g1**–**g3**) and also in agreement with the literature [37]. The CMC and γ_CMC_ depend on the molecular interaction at the interface of the water–micelle. Anything that facilitates the surfactant molecules to be adsorbed at the interface of the water–micelle will lead to a lowering in the surface tension. The presence of salts helped more closed packing at the interface by minimizing intermolecular repulsion and also promotes polar group adsorption at the air–water interface due to reduced hydration, which resulted in a lowering CMC [24,42]. In the comparison of the effect of different spacer groups on the surface properties, it was observed that the GS containing the diethyl ether spacer (**g1**) exhibited a lower CMC compared to those of **g2** and **g3**, and the CMC values were found in the order of **g2** ˃ **g3** ˃ **g1**, which indicates that **g1** is more prone to being adsorbed at the water–micelle interface.

## 4. Conclusions

In this work, three GSs (**g1**–**g3**) were synthesized for oilfield application and characterized by MALDI-ToF-MS, NMR, and FTIR. The influence of the hydrophilic spacer in thermal stability and aggregation morphologies was established. The GSs presented high solubility in deionized and saltwater due to the incorporation of polyethoxylates in the backbone of the chemical structure. The aqueous stability tests displayed that the **g1**–**g3** could maintain good competency at high salinity and high temperature conditions. The TGA showed high heat stability of all three GSs compared to the actual oilfield temperature and the GS with the secondary amine spacer (**g2**) exhibited comparatively higher heat stability with the retention of structural integrity up to 302 °C. It was established that the addition of salts moved the CMC and γ_cmc_ to smaller values in the following order DW ˃ NaCl ≈ SW. A substantial decline in CMC and γ_cmc_ was measured by moving from DW to NaCl. The GS containing the diethyl ether spacer (**g1**) was more prone to being adsorbed at the interface and formed a closely packed micelle structure that ultimately resulted in lowering the CMC. The CMC values of the GSs with different hydrophilic spacers were in the order of **g2** ˃ **g3** ˃ **g1**. The thermal and surface properties of the synthesized GSs show the practicability of using these surfactants for oilfield applications under harsh conditions.

## Figures and Tables

**Table 1:** 1H NMR readings describing the type of proton in **g1**–**g3** [1,28,36,37].

GSs	Proton NMR (δ in ppm, 500 MHz, CDCl_3_)									
	Lipophilic Tail			Ethoxy & Spacer	Acetic	Amide Group	Amido-Amine Group		Spacer	
	CH_3_ (a)	CH_2_ (b)	CH_2_ (c)	CH_2_ (d, i)	CH_2_ (e)	NH (f)	CH_3_ (j) CH_2_ (g)	CH_2_ (h)	CH_2_ (k)	-
**g1**	0.88	1.26	1.56	3.64	4.04	7.79	3.39	2.10	4.05	-
**g2**	0.88	1.26	1.57	3.68	4.00	7.82	3.44	2.11	3.44	2.91 (NH)
**g3**	0.87	1.26	1.56	3.65	4.00	7.93	3.41	2.07	4.04 (CH)	3.65 (OH)

**Table 2:** 13C NMR readings in **g1**–**g3**.

GSs	13C NMR (ppm, CDCl_3_, 125 MHz)
**g1**	13.9, 22.5, 25.9, 29.1, 29.3, 29.4, 31.7, 35.6, 51.7, 62.9, 63.3, 64.4, 70.3, 170.7
**g2**	14.1, 22.6, 26.0, 29.1, 29.3, 29.4, 31.9, 35.7, 51.7, 60.8, 61.5, 70.2, 170.9
**g3**	13.9, 22.5, 25.9, 29.1, 29.3, 29.4, 31.7, 35.8, 52.1, 60.6, 61.4, 70.3, 170.7

**Table 3:** FTIR readings showing adsorption values in **g1**–**g3**.

GSs	FTIR Characteristic Values (cm^−1^)								
	N-H	CH_2_ asym.	CH_2_ sym.	Amide (I)	Amide (II)	CH_2_ (Bending)	CH_3_ (Bending)	Ethoxy Stretch	asym.
**g1**	3424	2922	2855	1656	1544	1465	1349	1100	947
**g2**	3397	2922	2855	1655	1546	1466	1349	1099	947
**g3**	3412	2922	2855	1656	1544	1465	1349	1098	947

**Table 4:** MALDI-ToF-MS data of **g1**–**g3**.

GSs	Base Peak	Proposed Structure
**g1**	813.63	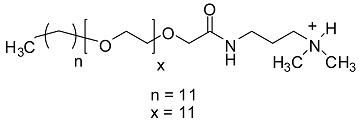
**g2**	725.57	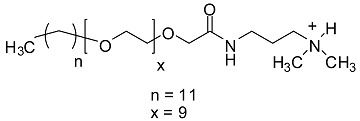
**g3**	681.54	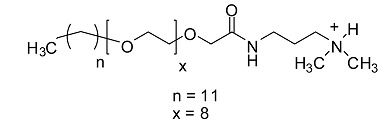

**Table 5:** The specific concentration of salt in SW and FW.

Ions	FW (g/L)	SW (g/L)
Na^+^	59.5	18.3
Ca^2+^	19.1	0.7
Mg^2+^	2.5	2.1
SO_4_^2−^	0.4	4.3
Cl^−^	132.1	32.2
HCO_3_^−^	0.4	0.1
Total	214	57.7

**Table 6:** Surface properties of **g1**, **g2**, and **g3** using different salinity conditions.

GSs	Solvent	CMC (mmol/L)	γ_cmc_ (mN/m)	г_max_ × 10^6^ (mol/m^2^)	A_min_ (nm^2^)
**g1**	DW	0.0049	39.64	3.66	0.45
**g1**	NaCl	0.0036	34.13	3.31	0.50
**g1**	SW	0.0029	34.66	3.17	0.52
**g2**	DW	0.0164	33.883	3.15	0.52
**g2**	NaCl	0.0123	32.914	2.63	0.63
**g2**	SW	0.0118	32.963	2.61	0.64
**g3**	DW	0.0102	34.72	3.28	0.51
**g3**	NaCl	0.0052	33.97	2.85	0.58
**g3**	SW	0.0064	33.65	3.12	0.52

## Supplementary Materials

The following are available online at https://www.mdpi.com/1996-1944/13/5/1046/s1, Figure S1: The comparison of 1H NMR before and after aging.
