# Supplementary material for: Poly(Oxyethylene)-Amidoamine Based Gemini Cationic Surfactants for Oilfield Applications: Effect of Hydrophilicity of Spacer Group
Source: Materials (Basel). 2020 Feb 26;13(5):1046. doi: 10.3390/ma13051046 (PMC7085195; doi:10.3390/ma13051046)
Supplement: Supplementary file 1 [file materials-13-01046-s001.pdf]

materials

Suppleme mtary Materials

Poly(Oxyethylene)-Amidoamine Based Gemini Cationic Surfactants for Oilfield Applications: Effect of Hydrophilicity of Spacer Group

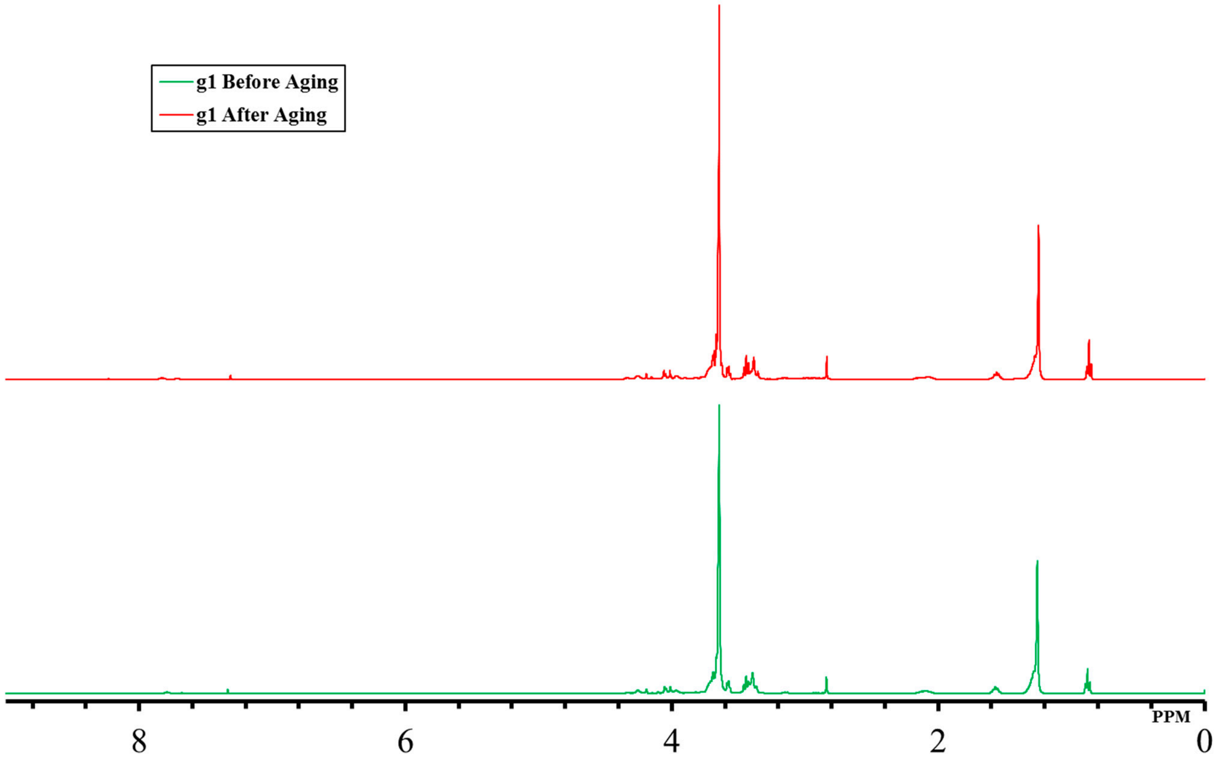

Figure S1. The comparison of 1H NMR before and after aging.
